# Using diverse U.S. beef cattle genomes to identify missense mutations in *EPAS1, *a gene associated with pulmonary hypertension

**DOI:** 10.12688/f1000research.9254.2

**Published:** 2016-10-05

**Authors:** Michael P. Heaton, Timothy P.L. Smith, Jacky K. Carnahan, Veronica Basnayake, Jiansheng Qiu, Barry Simpson, Theodore S. Kalbfleisch

**Affiliations:** 1 U.S. Meat Animal Research Center (USMARC), Clay Center, USA; 2 GeneSeek, a Neogen Company, Lincoln, USA; 3 Department of Biochemistry and Molecular Genetics, School of Medicine, University of Louisville, Louisville, USA

**Keywords:** Beef cattle, Whole genome sequence, EPAS1, HIF2A, Pulmonary hypertension, Brisket disease

## Abstract

The availability of whole genome sequence (WGS) data has made it possible to discover protein variants *in silico*. However, existing bovine WGS databases do not show data in a form conducive to protein variant analysis, and tend to under represent the breadth of genetic diversity in global beef cattle. Thus, our first aim was to use 96 beef sires, sharing minimal pedigree relationships, to create a searchable and publicly viewable set of mapped genomes relevant for 19 popular breeds of U.S. cattle. Our second aim was to identify protein variants encoded by the bovine endothelial PAS domain-containing protein 1 gene ( *EPAS1*), a gene associated with pulmonary hypertension in Angus cattle. The identity and quality of genomic sequences were verified by comparing WGS genotypes to those derived from other methods. The average read depth, genotype scoring rate, and genotype accuracy exceeded 14, 99%, and 99%, respectively. The 96 genomes were used to discover four amino acid variants encoded by *EPAS1 *(E270Q, P362L, A671G, and L701F) and confirm two variants previously associated with disease (A606T and G610S). The six *EPAS1* missense mutations were verified with matrix-assisted laser desorption/ionization time-of-flight mass spectrometry assays, and their frequencies were estimated in a separate collection of 1154 U.S. cattle representing 46 breeds. A rooted phylogenetic tree of eight polypeptide sequences provided a framework for evaluating the likely order of mutations and potential impact of *EPAS1* alleles on the adaptive response to chronic hypoxia in U.S. cattle. This public, whole genome resource facilitates *in silico* identification of protein variants in diverse types of U.S. beef cattle, and provides a means of translating WGS data into a practical biological and evolutionary context for generating and testing hypotheses.

## Introduction

The number of disease-causing mutations discovered in bovine genes has increased with the advancement of next-generation sequencing, reference genomes, and high density single nucleotide polymorphisms (SNPs) arrays. There are currently 130 Mendelian traits with known causal mutations in 117 cattle genes ^1^. The majority of these mutations cause disease by changing the gene’s protein sequence and thereby altering its normal function. As the list of major genes influencing bovine traits grows longer, there is increasing interest in the protein variants encoded by these genes, either in the source population, or in other uncharacterized populations. For example, in 2006 when a K211 mutation was discovered in the prion gene of a U.S. cow that developed atypical BSE, there was a pressing need to search for this potentially pathogenic allele in other U.S. cattle ^2, 3^. In principle, these searches can be accomplished *in silico* with access to population-scale gene sequence data.

Gene function can be affected by a wide range of genomic sequence differences including: large scale structural polymorphisms like translocations, inversions and copy number variants ^4^; and small scale differences like methylation, insertions/deletions (indels), and SNPs ^5^. Variants that alter amino acid sequences, such as missense, nonsense, frameshift, and splice site mutations, are among those most likely to affect function ^6^. These variants are readily identified by aligning genomic sequences of animals to an annotated reference genome assembly. An essential first step in understanding a gene’s potential influence on a trait, is determining whether any protein variants are encoded in a set of reference individuals ^7, 8^.

Access to population-scale gene sequence data, however, has been a limiting step for biomedical veterinary researchers studying U.S. cattle. The reagent costs for a traditional, single-gene approach with PCR-based Sanger exon resequencing of a population set can exceed tens of thousands of dollars, and take more than a year to complete ^9^. In addition, exon resequencing is a recurring cost for each gene analyzed. Approaches that use whole exome sequencing are less costly per gene, but incompletely target some bovine genes. Gene coverage with whole genome sequence (WGS) approaches is limited only by the quality of the reference genome, and the amount of data produced. WGS approaches have the advantage of high genotype accuracy and a low cost per gene, as the sequencing only needs to be accomplished once. The primary disadvantages of WGS approaches are the cost of production, the need for computing and informatics systems, and expertise for complex analyses. Regardless of the sequencing approach chosen, selecting the appropriate individuals for study, and verifying their identity and data integrity is essential.

Population-scale WGS data has been reported for a number of major cattle projects and is available at the National Center for Biotechnology Information (NCBI) through BioProjects and the Sequence Read Archive (SRA). Projects include 234 purebred cattle from global Holstein-Friesian, Fleckvieh, Jersey, and Angus breeds ^5^; and 379 Canadian cattle from six purebred beef breeds, three crossbred beef breeds, and the Holstein dairy breed ^10^. Key influential animals were chosen for these projects with the primary goal of using them to impute genetic variants and improve the accuracy of genomic prediction and genome wide association studies. However, these animals are not ideally suited for protein variant discovery across U.S. beef cattle populations, and the SRA data files are not readily searchable by gene. A third related population-scale WGS BioProject used 154 U.S. bulls from seven popular breeds and a mix of 116 crossbred and purebred animals from eight other breeds ^11^. However, the average genome coverage for these influential sires was 2.5 fold, and thus, insufficient for determining genotypes of individual animals.

The present report describes a publicly available and searchable set of mapped genomes for 96 beef sires from 19 breeds of U.S. cattle. These sires were chosen to minimize the relationships shared between pedigrees. The average read depth for these genomes was 14.8 and access is available without restrictions ^12^. The genome sequences may be either viewed directly with open source, high-performance visualization software ^13, 14^ via United States Department of Agriculture (USDA), Agricultural Research Service (ARS) internet sites, or downloaded from the SRA at NCBI. Visualization software, in combination with online access, allows users to navigate to a gene, visually identify, and accurately record protein variants occurring in U.S. beef cattle populations. Thus, if a gene mutation is reported in one breed, it can quickly be evaluated in other breeds, and searched for additional variants that may potentially affect protein structure.

The feasibility of this process was demonstrated for the bovine endothelial Per-ARNT-Sim (PAS) domain-containing protein 1 gene ( *EPAS1*) encoding the hypoxia inducible transcription factor 2A (HIF2A). *EPAS1* was selected for analysis because two linked missense mutations were reported to be associated with pulmonary hypertension (PH) in Angus cattle ^15^. PH causes right-sided heart failure (RHF) and has been recognized as an increasing problem in North American beef cattle in feedyards, and in dairy cattle ^16, 17^. Moreover, cattle treated for bovine respiratory disease complex (BRDC) in feedyard environments were three times more likely to die from RHF, and died earlier in the feeding period ^17^. The linked missense mutations in *EPAS1* encode threonine (T) and serine (S) at amino acid positions 606 and 610, respectively, and were associated with PH when compared to the more common allele encoding alanine (A) and glycine (G) at these positions ^15^. Animals carrying one or two copies of the *EPAS1* T606, S610 allele were significantly more likely to develop PH at high altitudes. Although other genes are likely involved, *EPAS1* appears to be a major gene affecting PH in cattle ^15^.

The protein encoded by *EPAS1* is one of three HIF-alpha proteins that plays an important role in transcriptional regulation of the hypoxic response in metazoans, and is highly conserved in mammals (reviewed in 18). HIF2A is an oxygen sensing subunit that forms a heterodimer with a common, constitutively expressed beta subunit, HIF1B. Each HIF heterodimer binds to specific hypoxia responsive elements and transcriptionally activates different sets of genes. HIF2A controls the chronic hypoxia response by binding small molecules, interacting with proteins, and binding to DNA. Amino acid substitutions in HIF2A have the potential to drastically affect those critical interactions.

In humans and mice, *EPAS1* missense mutations affecting the HIF2A oxygen-dependent degradation domain (ODDD) cause a gain-of-function, activate the hypoxia pathway, and can cause familial PH ^19, 20^. The *EPAS1* substitutions at positions 606 and 610 reported by Newman *et al.* are also in the ODDD of HIF2A, although not at the same positions as those in humans and mice ^15^. Although no additional *EPAS1* variants were previously observed in exons from the 20 Angus cattle sequenced ^15^, a broader reference set of U.S. beef cattle was not evaluated. Here, we report the discovery of four additional *EPAS1* missense mutations, a rooted phylogenetic tree of eight distinct HIF2A sequences, a genetic test for typing them, and HIF2A variant frequencies in a separate collection of 1154 U.S. cattle representing 46 breeds. The results illustrate the utility of the approach, and provide a resource for evaluating protein variants in specified genes of interest. Knowledge of cattle protein variants affecting function is critical for transitioning from a descriptive phase of genomics to an applied phase where animal health, welfare, and production may be improved.

## Methods

### Ethics statement

This article contains no studies performed with animal subjects. Archival DNA was used from extracts of samples that were either: purchased from commercial sources that collected them for artificial insemination of cattle and not for research, purchased from individuals that collected them privately for their purposes (such as food), or donated to the U.S. Meat Animal Research Center (USMARC) by private individuals that collected them privately for their own purposes.

### Discovery and validation panels of cattle

**Figure 1.**
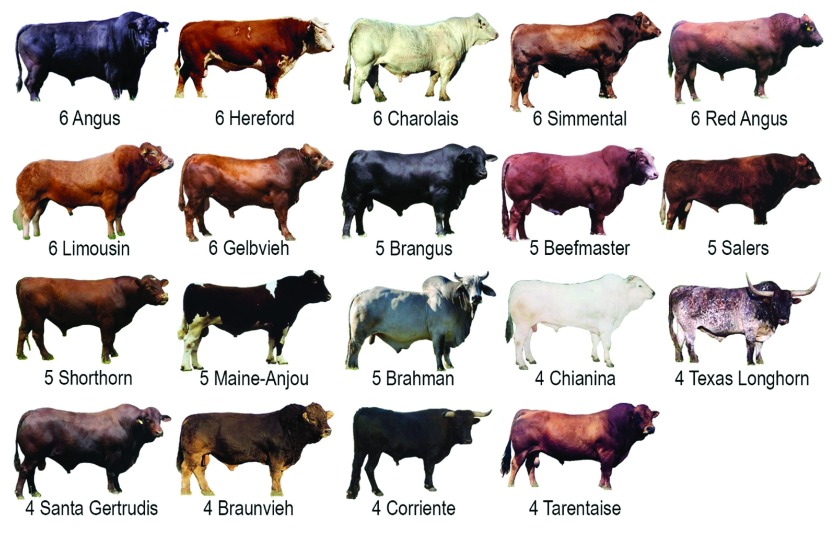
USMARC Beef Cattle Diversity Panel version 2.9. This group of 96 registered beef sires was chosen to have a minimum of pedigree relationships within each of the 19 breeds.

The discovery panel consists of 96 unrelated individuals from 19 popular U.S. beef breeds (USMARC Beef Diversity Panel version 2.9 [MBCDPv2.9], Figure 1). The current panel design was based on a previous set of commercially-available sires from 16 breeds with minimal pedigree relationships (MBCDPv2.1) ^21^. For both panels, pedigrees were obtained from leading suppliers of U.S. beef cattle semen and analyzed to identify unrelated individuals for inclusion. On the basis of the number of registered progeny, the breeds in the MBCDPv2.1 were estimated to represent greater than 99% of the germplasm used in the US beef cattle industry, contain more than 187 unshared haploid genomes, and allow a 95% probability of detecting any allele with a frequency greater than 0.016 ^21^. As previously described, this “threshold” frequency was defined as the minimum allele frequency at which the probability of observing the allele at least once in an animal group was 0.95. The probability of observing an allele at least once is 1 − (1 − p) ^n^ where “p” is the frequency of the allele and “n” is the number of independent samplings, or, in this case, the number of unshared haploid genomes for diploid organisms. This assumes that samplings (haploid genomes) are independent and identically distributed (the same p applies to all animals). Setting power or the probability of observing the allele at least once to 0.95 results in the equation: 0.95 = 1 − (1 − p) ^n^. Solving this equation for p yields p = 1 − (0.05) ^1/n^ for all p between 0 and 1. The panel was updated to increase the number of beef breeds from 16 to 19, and remove the Holstein breed which was well represented in other WGS datasets. To make room for three additional beef breeds (Braunvieh, Corriente, and Tarentaise), the maximum number of sires within a breed was reduced from eight to six (NCBI BioProject PRJNA324822).

A separate set of cattle samples was used to validate results obtained from the above discovery panel. The validation panel consisted of samples from male and female registered purebred cattle with diverse pedigrees. Samples were from semen, blood, or hair follicles, depending on gender and availability as previously described ^3^. Where possible, animals within breed were chosen so they did not share parents or grandparents, and none were closely related to the 96 sires in the MBCDPv2.9. The breeds and samples used were: Angus (n = 24), Ankole-Watusi (n = 24), Ayrshire (n = 24), Beefmaster (n = 24), Belgian Blue (n = 24), Blonde d'Aquitaine (n = 24), Brahman (n = 24), Brahmousin (n = 24), Braunvieh (n = 24), Brangus (n = 24), Brown Swiss (n = 26), Charolais (n = 24), Chianina (n = 24), Corriente (n = 24), Devon (n = 24), Dexter (n = 24), Gelbvieh (n = 24), Guernsey (n = 25), Hereford (n = 24), Highland (n = 24), Holstein (n = 86), Indu-Brazil (n = 24), Jersey (n = 28), Limousin (n = 24), Maine-Anjou (n = 24), Marchigiana (n = 24), Mini-Hereford (n = 24), Mini-Zebu (n = 24), Montbeliarde (n = 24), Murray Grey (n = 21), Nelore (n = 24), Piedmontese (n = 24), Pinzgauer (n = 24), Red Angus (n = 24), Red Poll (n = 24), Romagnola (n = 24), Salers (n = 24), Santa Gertrudis (n = 24), Senepol (n = 23), Shorthorn (n = 24), Simmental (n = 24), Tarentaise (n = 24), Texas Longhorn (n = 24), Texas Longhorn, Cattlemen’s Texas Longhorn Registry (CTLR, n = 22), Tuli (n = 24), and Wagyu (n = 24).

### WGS production, alignment, and SNP genotyping

DNA was extracted from commercial semen with a typical phenol:chloroform method and stored at 4°C in 10 mM TrisCl, 1 mM EDTA (pH 8.0) as previously described ^22^. Approximately 5 μg of bovine genomic DNA was fragmented by focused-ultrasonication to generate fragments less than 800 bp long (Covaris, Inc. Woburn, Massachusetts USA). These fragments were used to make an indexed, 500 bp paired-end library according to the manufacturer’s instructions (TruSeq DNA PCR-Free LT Library Preparation Kits A and B, Illumina, Inc., San Diego, California USA). After construction, indexed libraries were pooled in groups of four to eight, and sequenced with a massively parallel sequencing machine and high-output kits (NextSeq500, two by 150 paired-end reads, Illumina Inc.). After sequencing, the raw reads were filtered to remove adaptor sequences, contaminating dimer sequences, and low quality reads. Pooled libraries with compatible indexes were repeatedly sequenced until 40 GB of data with greater than Q20 quality, was collected for each sire. In preliminary trials, 40 GB of Q20 data consistently resulted in greater than 10-fold read coverage for each animal. Previous results showed that this level of coverage provided scoring rates and accuracies that exceeded 99% ^23^.

The DNA sequence alignment process was similar to that previously reported ^23^. Briefly, FASTQ files corresponding to a minimum of 40 GB of Q20 sequence were aggregated for each animal. DNA sequences from FASTQ files were aligned individually to UMD3.1 ^24^ with the BWA aln algorithm version 0.7.12 ^25^, then merged and collated with bwa sampe. The resulting sequence alignment map (SAM) files were converted to binary alignment map (BAM) files, and subsequently sorted via SAMtools version 1.3.1 ^26^. Potential PCR duplicates were marked in the BAM files using the Genome Analysis Toolkit (GATK) version 3.6 ^27^. Regions in the mapped dataset that would benefit from realignment due to small indels were identified with the GATK module RealignerTargetCreator, and realigned using the module IndelRealigner. The BAM files produced at each of these steps were indexed using SAMtools. The resulting indexed BAM files were made immediately available via the Intrepid Bioinformatics genome browser http://www.intrepidbio.com/ with groups of animals linked at the USMARC WGS browser http://www.ars.usda.gov/Services/Docs.htm?docid=25585. The raw reads were deposited at NCBI BioProject PRJNA324822. Mapped datasets for each animal were individually genotyped with the GATK UnifiedGenotyper with arguments “--alleles” set to the VCF file ( File S1), “--genotyping_mode” set to “GENOTYPE_GIVEN_ALLELES”, and “--output_mode” set to “EMIT_ALL_SITES”. Lastly, some SNP variants were identified manually by inspecting the target sequence with IGV software version 2.1.28 (described in the Methods section entitled ‘Identifying protein variants encoded by *EPAS1’*). In these cases, read depth, allele count, allele position in the read, and quality score were taken into account when the manual genotype determination was made.

### Evaluating WGS data integrity with 121 reference SNPs and 770 k bead array SNPs

Genotypes from a set of 121 reference SNPs were used as an initial verification of the WGS datasets. Many of these DNA markers have been widely used for parentage determination, animal identification, and disease traceback ( Table S1) ^21, 28, 29^. The 121 reference SNPs were previously genotyped across the MBCDPv2.9 by multiple PCR-Sanger sequencing reactions, two independent designs of multiplexed matrix-assisted laser desorption/ionization time-of-flight mass spectrometry (MALDI-TOF MS) genotyping assays, and multiple bead array platforms, and are tabulated in Table S2. The error rate in the WGS data was estimated by comparing the consensus genotypes for these SNPs to the WGS genotypes. An animal’s WGS dataset passed initial verification when the accuracy of the WGS genotypes exceeded 97%, and the average mapped read depth was proportional to the amount of WGS data collected. Animals’ datasets that failed this initial verification were inspected closely for contaminating and/or missing files. Electronic file transfer errors resulted in contaminated and missing data for approximately one third of the 96 WGS datasets and required systematic testing, correction, and reprocessing. Linear regression analysis was accomplished in Excel version 2016. Access to the sequence via USDA internet site ( http://www.ars.usda.gov/Services/Docs.htm?docid=25585) and Intrepid Bioinformatics site ( http://server1.intrepidbio.com/FeatureBrowser/customlist/record?listid=7686214634) was provided as soon as the .BAM files were produced. Because the raw datasets were available online as they were produced, the FASTQ files were deposited in the NCBI SRA only after they were validated as described above. These 96 sets of files can be accessed through BioProject PRJNA324822 in the Project Data table under the Resource Name: SRA Experiments. SNPs from the BovineHD BeadChip (Illumina Inc.) were selected for comparison because they were numerous, uniformly distributed across the bovine genome, and available. Based on the nucleotide sequence of the probes obtained from the manufacturer, the positions of the SNPs were verified via a BLAT process as previously described ^23^. A total of 772,990 variant positions were successfully mapped with this process, with 54 positions being discrepant when compared to those in the manufacturer’s most recent release of probe descriptions. The VCF file for these 772,990 variants is provided ( File S1). The genotypes from the WGS data were compared to those from the high-density bead array with a custom program written specifically for this operation. Three classes of discordant genotypes were identified. First, were those scored as homozygous in the WGS data and heterozygous in bead array data. These could have resulted from low coverage in WGS data at that position, or errors in the bead array caused by probes hybridizing to repeated sequences. The second type of discordance was scored as heterozygous in the WGS data, and homozygous in the bead array data. These could have resulted from allele-specific probe hybridization problems in the bead array platform. The final category consisted of missing genotypes in the bead array data, which were likely caused by errors in the conversion of the manufacturer’s “AB” genotype calls to the nucleotide calls.

### Identifying protein variants encoded by bovine *EPAS1*

Using public internet access to USMARC sites, the nucleotide variation in the exon regions of *EPAS1* was visualized with open source software installed on a laptop computer and recorded manually in a spreadsheet. Briefly, a Java Runtime Environment (Oracle Corporation, Redwood Shores, CA) was first installed on the computer. When links to the data were selected from the appropriate web page, IGV software ^13, 14^ automatically loaded from a third-party site (Intrepid Bioinformatics, Louisville KY) and the mapped reads were loaded in the context of the bovine UMD3.1 reference genome assembly. For viewing *EPAS1* gene variants, WGS from a set of eight animals of different breeds was loaded (“mixed groups of 8”, http://www.ars.usda.gov/Research/docs.htm?docid=25586) and the IGV browser was directed to the appropriate genome region by entering “EPAS1” in the search field. The IGV zoom function was used to view the first exon at nucleotide resolution with the “Show translation” option selected in IGV. The exon sequences were visually scanned for polymorphisms that would alter amino acid sequences, such as missense, nonsense, frameshift, and splice site mutations. Once identified, the nucleotide position corresponding to a protein variant was viewed and recorded for all 96 animals. Using IGV, codon tables, and knowledge of the HIF2A protein sequence (NP_777150), the codons affected by nucleotide alleles were translated into their corresponding amino acids and their positions noted. Haplotype-phased protein variants were assigned unambiguously in individuals that were homozygous, and those individuals with only one variant amino acid. A maximum parsimony phylogenetic tree was constructed manually from the unambiguously phased protein variants and used to infer phase in any remaining variants with simple maximum parsimony assumptions.

WGS datasets from five closely-related Bovinae species were mapped to the cattle reference assembly UMD3.1 with a process similar to that previously reported ^23^. These mapped Bovinae samples included two each of yak, gaur, and banteng; and one sample each of plains bison, water buffalo. The mapped genomes were visually inspected across the *EPAS1* exons in the same browser environment as the cattle data, and variant codons were recorded. Information about the source and the content of the WGS datasets is provided in Table S3. Because reference SNP genotypes are not readily available for these species, verification of the integrity and quality of the newly sequenced Bovinae WGS datasets was limited. For each dataset, the mapped read density in conserved exons was estimated and compared to the amount of Q20 sequence collected for that animal. No inconsistencies were noted between the expected and observed read depths. In addition, distinctive homozygous “species-specific” nucleotides were observed for each species, and these same nucleotides were not observed in the other species. The genomes for all eight animals were made viewable by IGV at http://www.ars.usda.gov/Services/Docs.htm?docid=25585. They are also available at NCBI BioProjects: PRJNA325061, PRJNA221623, and PRNJA207334.

### MALDI-TOF MS genotyping of six *EPAS1* missense mutations

A single multiplex assay was designed for the six *EPAS1* missense SNPs with the information in Table 1 with software provided by the manufacturer (Agena Biosciences, San Diego, California, USA). The oligonucleotide sequences and assay conditions are provided in Table S4. After design and validation with bovine control DNAs for each SNP, the MBCDPv2.9 DNA was tested in a blinded experiment in which the true genotypes were unknown by those typing the samples. Assay design and genotyping was performed at GeneSeek (Lincoln, Nebraska, USA) with the MassARRAY platform and iPLEX Gold chemistry according to the manufacturer’s instructions (Agena Biosciences). MALDI-TOF MS genotypes for six SNPs are provided for the MBCDPv2.9 and 1154 of 1168 cattle from 46 breeds in Table S5.

**Table 1.** DNA sequence information for bovine *EPAS 1* missense mutations identified in the beef cattle diversity panel (MBCDPv2.9).

Codon variant ^a^	Position (UMD3.1)	Exon	HIF2A domain ^b^	Consensus codon sequence ^c^	Codon alleles ^d^	MAF ^e^	Flanking genomic sequence
E270Q	chr11: 28650973	7	PAS-B	**S**aa	**G**aa = E **C**aa = Q	0.094	ttttttttttttttcaatttagaatcacagaactggttggttaccaccct **[S]**aagagctgcttggccgctcagcctatgagttctaccatgcactggactca
P362L	chr11: 28659040	9	ID	c **Y**g	c **C**g = P c **T**g = L	0.094	tgaaattgagaagaacgacgtggtgttctccatggatcagacagagtcac **[Y]**gtttaagccgcacctgctgaccatgaacagcatctttgataacagtggca
A606T ^f^	chr11: 28662654	12	ODDD	**R**cc	**G**cc = A **A**cc = T	0.068	agcagctggaaagcaagaagacggagcctgagcagcggcgtgtgtccttc **[R]**ccttctttgacRgtgggagcagggtgtccctgctgcagtgctgtggtcag
G610S ^f^	chr11: 28662666	12	ODDD	**R**gt	**G**gt = G **A**gt = S	0.068	gcaagaagacggagcctgagcagcggcgtgtgtccttcRccttctttgac **[R]**gtgggagcagggtgtccctgctgcagtgctgtggtcagacctacaccccc
A671G	chr11: 28662850	12	ID	g **S**c	g **C**c = A g **G**c = G	0.036	agaccggcacgcagaggccgtgggggcagcgcccctggggctcccccccg **[S]**cacaccccatctcgccatgctcaagaagaggtcagtgatggagatgctgg
L701F	chr11: 28663897	13	ID	**Y**tc	**C**tc = L **T**tc = F	0.005	agggcttcgggcctcagggtccagacgtgatgagcccagccatgattgcc **[Y]**tctccaacaagctgaagctgaagcgacagctggagtacgaggagcaagcc

^a^The bovine *EPAS1* gene is oriented in the sense direction with regards to the UMD3.1 reference assembly. All sequences presented are from the sense strand. ^b^HIF2A protein domain abbreviations: PAS-B, Per-Arnt-Sim domain B; ID, interdomain; and ODDD, oxygen-dependent degradation domain. ^c^IUPAC/IUBMB ambiguity codes used for nucleotides: R = a/g, Y = c/t, M = a/c, K = g/t, S = c/g, W = a/t ^40^. ^d^The major allele is listed first. ^e^Minor allele frequency in MBCDPv2.9 ^f^Missense mutations associated with high altitude PH ^15^.

## Results

### Panel design, genome sequencing, and quality control of WGS datasets

A beef cattle diversity panel was designed to broadly sample the genetic diversity of U.S. populations, while fitting within the constraints of a 96-sample format, often used for automated DNA sequencing and genotyping. The composition and design of the panel was updated from a previously reported set as described in the Methods. A minimum of four sires were included for each breed, with the more popular U.S. breeds having five or six animals ( Figure 1). There was relatively little power for detecting rare variants within breed, since not more than 12 haploid genomes were sampled (95% probability of detecting any polymorphism with a frequency greater than 0.22, Methods). Despite the modest power within breed, sequencing the entire panel significantly increased the chances of detecting relatively rare variants segregating in U.S. beef cattle. With more than 187 of 192 unshared haploid genomes in the 96 sires, it was estimated there was a 95% probability of observing polymorphisms with a frequency greater than 0.016. Thus, the power for allele detection in this beef diversity panel was derived from having exceedingly few pedigree relationships within breed, and essentially none between breeds.

The WGS was generated by sequencing indexed pools of libraries whose composition was adjusted iteratively across multiple instrument runs to achieve at least 40 GB of FASTQ sequence. The average amount of total sequence per sample was 48.3 GB (±12.0) and varied between 40.2 GB and 109.4 GB. This approach reduced the overall data production cost, however each animal had data files from multiple sequencing runs that required manual collation prior to analysis, and thus increased the labor cost. In addition to the usual challenges of sample contamination, sample switches, missing data, variable quality data, and data transfer errors, the FASTQ files produced by the instrument had identical names across multiple machine runs. This added another layer of complexity to maintaining file provenance. The process of manually aggregating and transferring an average of 42 similarly-named FASTQ files for each animal was inherently prone to error and unavoidable with the instrument and the institutional network security restrictions.

**Figure 2.**
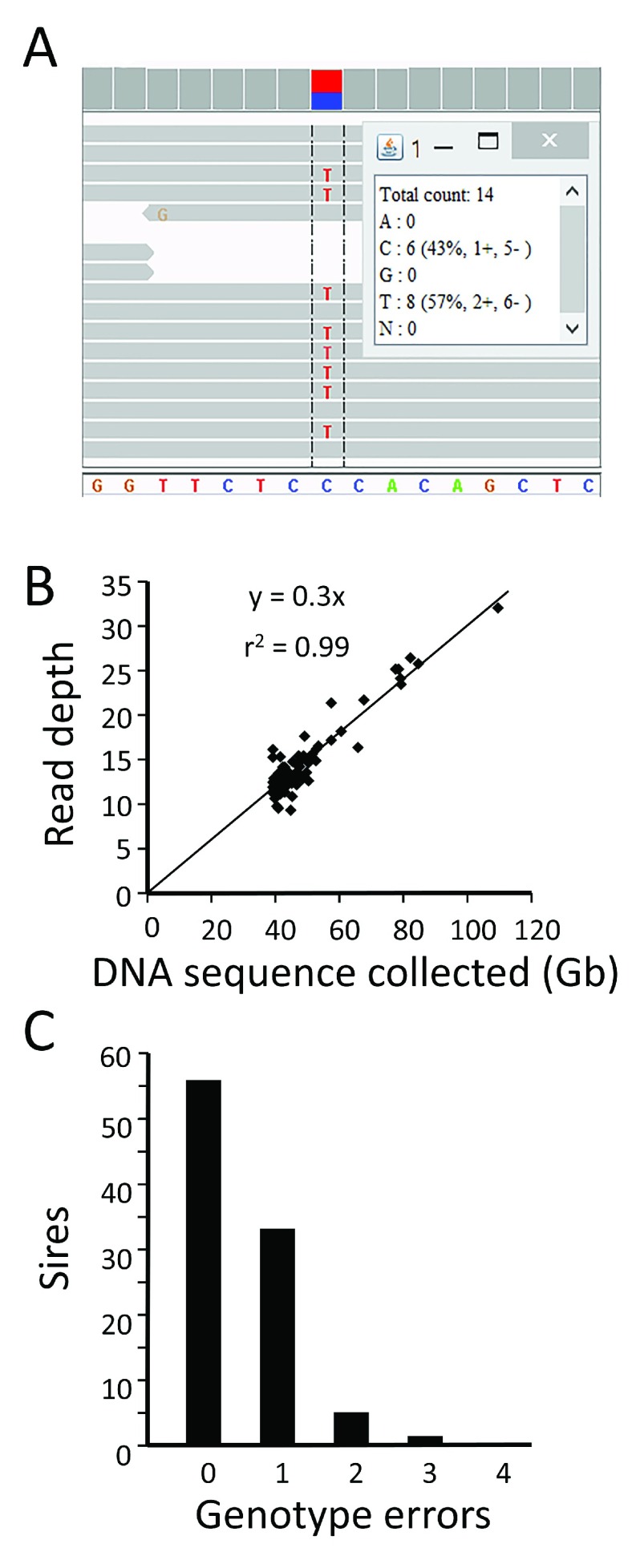
Comparison of 121 reference SNP genotypes with those derived from WGS data. Panel **A**: Computer screen image of one animal’s WGS data aligned to bovine reference assembly UMD3.1 at a reference SNP site. The heterozygous C/T genotype is shown as viewed with the IGV software ^13, 14^. Panel **B**: Linear relationship between mapped read depth and the amount (Gb) of Q20 WGS data collected. At each SNP position, the read depth and genotypes were visualized and manually recorded for 121 parentage SNPs. A list of these 121 parentage SNPs and their sequence information is provided in Table S1. Panel **C**: Genotype scoring accuracy for 121 parentage SNPs in 96 sires. Consensus reference genotypes (n = 11,616) for the parentage SNPs were previously determined by multiple methods ( Table S2).

Thus, to verify the WGS data integrity at the end of the process, genotypes from a set of 121 reference SNPs were used as a first test. These SNPs are distributed across the genome, highly-informative in U.S. beef cattle, have been widely used for bovine parentage testing (Methods). The WGS-derived genotypes for these 121 SNPs were obtained by viewing an animal’s mapped reads at the relevant genome coordinates, with public software, a third party database, and web links created for this task (illustrated in Figure 2A, http://www.ars.usda.gov/Research/docs.htm?docid=25586). As described in the Methods, data inconsistencies of multiple types were discovered by comparison with the known reference genotypes and corrected in approximately one third of the file sets. Comparison to the reference SNP genotypes also provided a check for the expected linear relationship between the amount of sequence collected and the depth of reads mapped to the reference assembly. Regression analysis showed that the average read depth at the 121 reference SNPs was directly proportional the amount of sequence collected ( Figure 2B). The 48.3 GB of sequence collected for each animal resulted in an average of 14.4-fold depth of mapped read coverage. The overall accuracy of WGS genotypes for the 121 reference SNPs was 99.5%, with 56 sires having 100% concordance ( Figure 2C). The few WGS genotype errors observed were typically caused by undetected heterozygous alleles at sites with low read coverage. Thus, the use of 121 reference SNPs was effective for discovering and repairing errors in these WGS datasets, and verifying the coverage.

**Figure 3.**
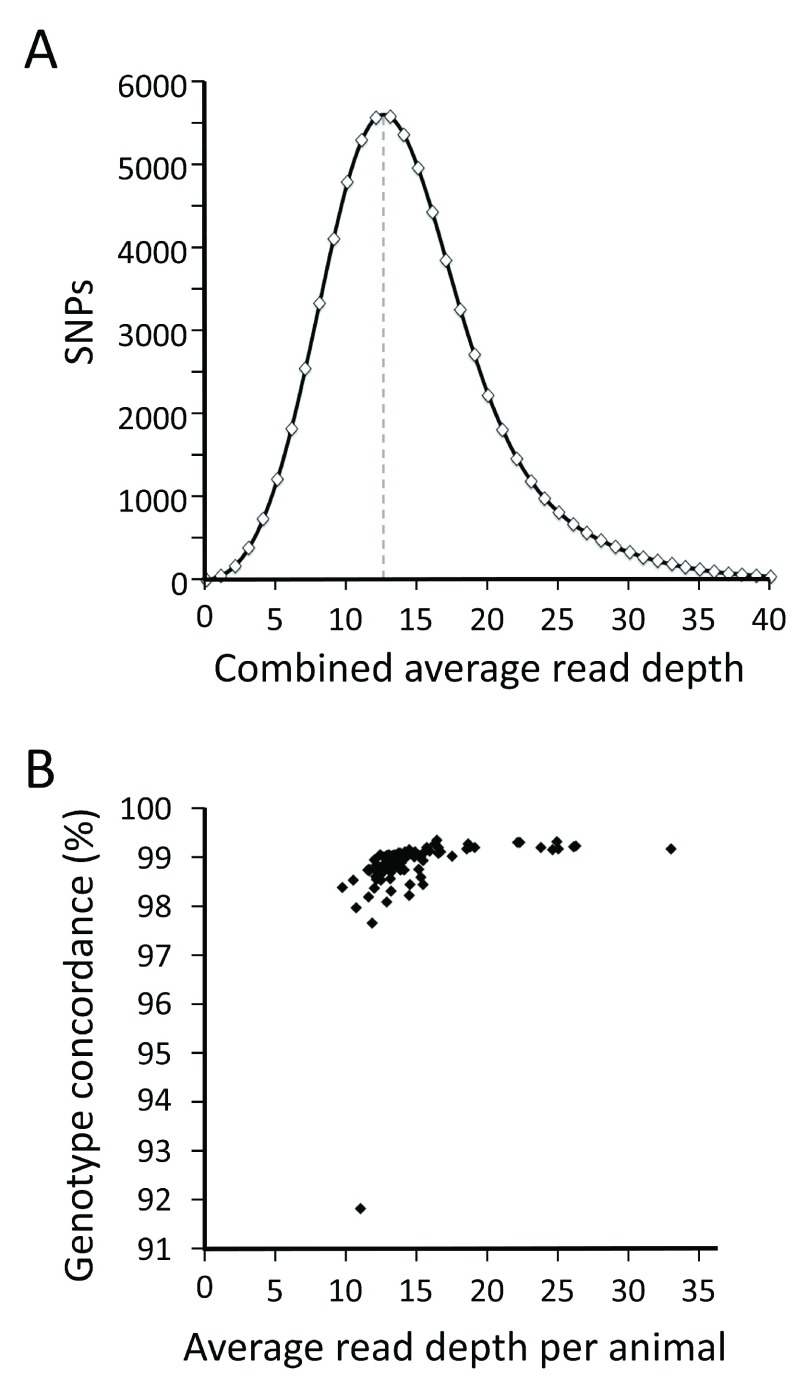
Comparison of WGS genotypes from 96 sires with those from bead arrays. Panel **A**: The distribution of average WGS read depth across 730 k SNP sites for 96 sires combined. Panel **B**: A comparison of the average WGS read depth per animal to the average genotype concordance between 730 k WGS and bead array genotypes.

A broader characterization of the coverage and quality of each dataset was accomplished by comparing an average of 730,410 of SNP genotypes from each sire to those from a high density bead array (Methods). The average distribution of read depths was slightly positively skewed with a mode of 12.5 when combined for all animals ( Figure 3A). The average read depth for these 730 k SNPs (14.8) was in close agreement with that for the 121 reference SNPs (14.4), confirming that the smaller SNP set was not biased subset of the larger set. Averaged over all animals, the concordance between WGS genotypes and those from bead arrays was high (98.8%, Figure 3B) and also agreed well with results from the 121 reference SNPs (99.5%). A surprising feature of this analysis was that the genotype concordance reached a maximum at approximately 99%, in spite of increasing coverage. Thus, WGS datasets with 13-fold and 33-fold coverage had 99.1 and 99.2 % concordance, respectively, possibly reflecting the percentage of bead array genotypes with problems. One notable exception was Corriente sire 19202900 which had a concordance of 91.8% ( Figure 3B). However, the 121 reference SNP genotypes for this same animal were 98.4% accurate (119/121). This result suggests that the lower genotype concordance in the Corriente sire may have been caused by the quality of the bead array data. For all other animals, the discordant genotypes were infrequent, with “allele dropouts” being the most common type (Methods). Allele dropouts were inferred at a SNP site when one allele of a heterozygote was not detected (i.e., “dropped”). Although rare, there were more dropped alleles observed in the bead array data (1.1%) than for the WGS data (0.7%). Taken together, the analyses indicate that the WGS datasets from these 96 diverse beef sires are of sufficient quality and coverage for use in identifying and decoding gene variants in U.S. beef cattle.

### Identification of protein variants encoded by *EPAS1*

**Figure 4.**
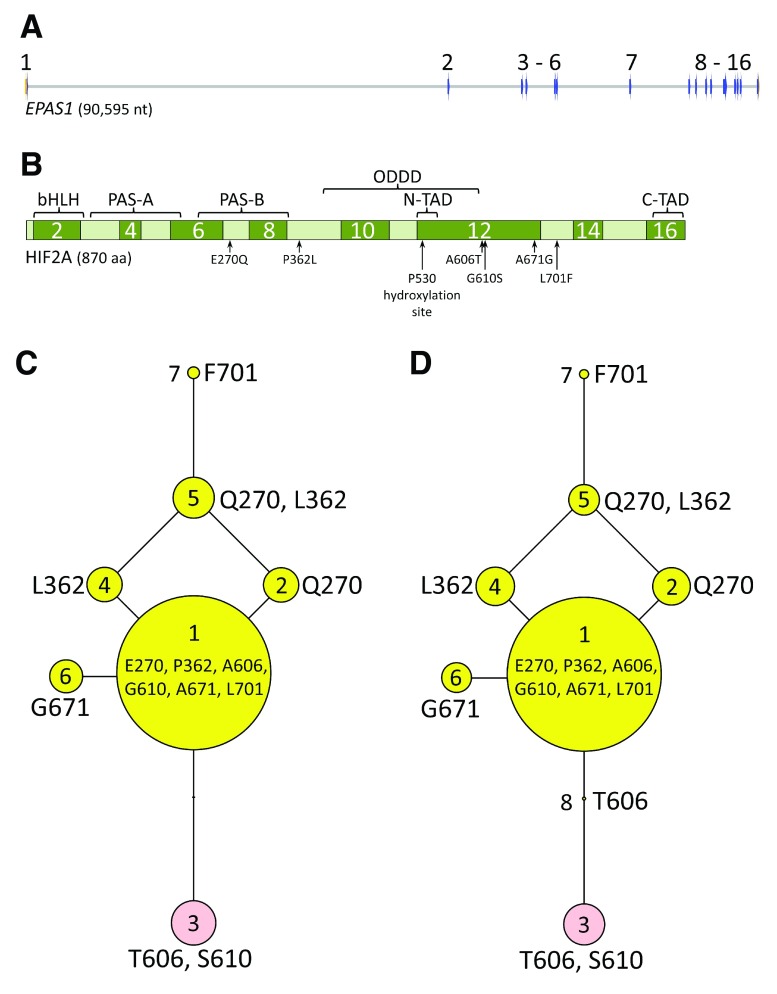
Physical maps and unrooted maximum parsimony phylogenetic trees of HIF2A protein variants found in cattle. Panel **A**, genomic DNA map of *EPAS1*: blue arrows, exon regions; grey horizontal lines, intron regions. Panel **B**, map of HIF2A domains in relationship to missense mutations found in cattle: bHLH, basic helix-loop-helix domain; PAS-A and PAS-B, Per-Arnt-Sim domains; ODDD, oxygen-dependent degradation domain; N-TAD, N-terminal transactivation domain; C-TAD, C-terminal transactivation domain. Panels **C** and **D** represent results from the 96-member, 19 breed diversity panel and the 1154-member, 46 breed set, respectively. The most frequent HIF2A isoform (“variant 1”) was used as the reference sequence for the trees. For “variants 1” through “8”, each node in the tree represents a different isoform of HIF2A that varies by one amino acid compared to adjacent nodes. The areas of the circles are proportional to the variant frequency in the group of cattle tested. “Variant 3” (pink circle; T606, S610) is identical to that associated with PH in Angus cattle ^15^. “Variant 2” (Q270) is identical to the 870 amino acid protein encoded by the bovine reference assembly UMD3.1.

The 96 sets of aligned WGS data were visually analyzed in the *EPAS1* coding region to identify potential HIF2A protein variants (Methods). *EPAS1* consists of 16 exons spanning 90 kb of genomic DNA and encodes an 870 amino acid protein with multiple functional domains ( Figure 4A and Figure 4B). Viewing the aligned sequences and detecting variants was simple, fast, and accurate with the IGV software and a browser developed for this purpose ( Figure S1). Four previously undescribed missense mutations were discovered and predicted to cause the substitution of glutamine (Q) for glutamate (E) at position 270; leucine (L) for proline (P) at position 362; glycine (G) for Alanine (A) at position 671; and phenylalanine (F) for leucine (L) at position 701 ( Table 1 and Figure 4B). The two additional amino acid variants previously associated with PH, were also observed (A606T and G610S). No other missense, nonsense, frameshift, splice site, or indel variants affecting the coding region were detected. Haplotypes encoding seven predicted HIF2A variants were translated and placed in the context of a phylogenetic tree ( Figure 4C). Five of seven predicted HIF2A protein variants (variants “2”, “4”, “5”, “6”, and “7”) were previously unreported, and accounted for 17% of the total in the beef cattle diversity panel.

To verify the accuracy of *EPAS1* genotypes and determine the protein variant frequencies in a larger set of U.S. cattle, MALDI-TOF MS assays were developed for the six missense SNPs (Methods). In a blinded test, 575 of 576 (99.8%) *EPAS1* MALDI-TOF MS genotypes from the 96 sires were concordant with those from WGS, confirming that the newly discovered SNPs were authentic and the WGS and MALDI-TOF MS genetic tests were accurate. The average HIF2A variant frequencies in a set of 1154 purebred cattle from 46 breeds were similar to those observed in the beef cattle diversity panel ( Table 2 and Figure 4D) with a call rate of 98.8%.

**Table 2.** Frequencies of predicted HIF2A protein variants in U.S. cattle.

Protein variant code ^a^	Variant amino acids ^b^	Protein variant frequency ^c^	
		Beef cattle diversity panel (n = 96)	Additional purebred cattle (n = 1154)
1	E270, P362, A606, G610, A671, L701	0.760	0.782
2	**Q270**, P362, A606, G610, A671, L701	0.042	0.052
3	E270, P362, **T606**, **S610**, A671, L701	0.068	0.054
4	E270, **L362**, A606, G610, A671, L701	0.042	0.048
5	**Q270**, **L362**, A606, G610, A671, L701	0.047	0.031
6	E270, P362, A606, G610, **G671**, L701	0.036	0.030
7	**Q270**, **L362**, A606, G610, A671, **F701**	0.005	0.000
8	E270, P362, **T606**, G610, A671, L701	0.000	0.003

^a^HIF2A protein variant allele definitions are shown in Figure 4. ^b^The bolded residues are those differing from “variant 1”. ^c^The coefficient of determination for these frequencies (r ^2^) was 99.9

The HIF2A isoform associated with an increased risk for PH in Angus cattle (T606, S610; “variant 3”) was observed in 18 of 46 breeds, with four breeds having frequencies higher than Angus ( Table 3). The Guernsey dairy breed had the highest proportion of the risk allele with 18 of 26 animals (69%) having one or two copies of “variant 3” ( Table S5). Notably, all 96 animals from the *Bos indicus* breeds (Brahman, Nelore, Indu-Brazil, and mini-zebu) were homozygous for the most common HIF2A “variant 1”. An important result of typing the extended 46 breed set of cattle, was the discovery of an unlinked T606 mutation (“variant 8”, Figure 4D) present in Romagnola, Chianina, and Maine-Anjou cattle ( Table S5). The discovery of an eighth variant brought the number of possible HIF2A diploid combinations to 36, and underscored the importance of accurate HIF2A typing in animals used to study PH and RHF in beef cattle.

**Table 3.** The frequencies of predicted HIF2A protein variants in 46 U.S. breeds.

Breed group	Animals typed	HIF2A protein variant allele frequency ^a^							
		1	2	3 ^b^	4	5	6	7	8
Angus	23	0.76	-	**0.22**	-	-	0.02	-	-
Ankole-Watusi	24	0.94	-	-	0.06	-	-	-	-
Ayrshire	24	0.31	-	**0.13**	0.19	-	0.38	-	-
Beefmaster	24	0.92	0.02	-	-	-	0.06	-	-
Belgian Blue	24	0.65	0.29	-	0.04	0.02	-	-	-
Blonde d'Aquitaine	23	0.89	0.02	-	0.04	0.04	-	-	-
Brahman	24	1.00	-	-	-	-	-	-	-
Brahmousin	24	0.94	-	**0.02**	-	0.04	-	-	-
Brangus	23	0.57	0.17	**0.26**	-	-	-	-	-
Braunvieh	24	0.67	-	-	0.23	0.10	-	-	-
Brown Swiss	26	1.00	-	-	-	-	-	-	-
Charolais	24	0.77	0.04	**0.06**	-	-	0.13	-	-
Chianina	24	0.75	0.13	**0.10**	-	-	-	-	0.02
Corriente	24	0.67	-	-	0.31	0.02	-	-	-
Devon	23	0.96	0.04	-	-	-	-	-	-
Dexter	24	0.71	0.08	**0.15**	0.02	0.04	-	-	-
Gelbvieh	24	0.52	-	-	0.33	0.15	-	-	-
Guernsey	26	0.42	-	**0.52**	0.06	-	-	-	-
Hereford	24	0.63	0.02	**0.04**	-	-	0.31	-	-
Highland	24	0.83	0.04	**0.08**	-	0.04	-	-	-
Holstein	80	0.72	0.19	-	0.04	0.04	-	-	-
Indu-Brazil	24	1.00	-	-	-	-	-	-	-
Jersey	28	0.88	0.09	-	0.04	-	-	-	-
Limousin	24	0.85	-	**0.02**	0.06	0.06	-	-	-
Maine-Anjou	24	0.81	0.06	**0.02**	-	0.08	-	-	0.02
Marchigiana	24	0.98	0.02	-	-	-	-	-	-
Mini Hereford	24	0.60	-	-	-	-	0.40	-	-
Mini Zebu	24	1.00	-	-	-	-	-	-	-
Montbeliarde	24	0.67	-	-	0.08	0.25	-	-	-
Murray Gray	21	0.67	0.02	**0.24**	-	0.02	0.05	-	-
Nelore	24	1.00	-	-	-	-	-	-	-
Piedmontese	24	0.98	0.02	-	-	-	-	-	-
Pinzgauer	24	0.83	0.02	-	0.13	0.02	-	-	-
Red Angus	24	0.60	0.06	**0.33**	-	-	-	-	-
Red Poll	24	0.58	-	**0.21**	-	0.21	-	-	-
Romagnola	24	0.90	-	-	-	-	-	-	0.10
Salers	23	0.74	0.02	-	0.11	0.13	-	-	-
Santa Gertrudis	24	0.69	0.29	-	-	-	0.02	-	-
Senepol	23	0.87	0.02	**0.07**	-	0.04	-	-	-
Shorthorn	24	0.79	0.15	-	-	0.04	0.02	-	-
Simmental	24	0.90	-	**0.04**	-	0.02	0.04	-	-
Tarentaise	24	0.65	-	**0.10**	0.19	0.02	0.04	-	-
Texas Longhorn	24	0.85	0.08	-	0.06	-	-	-	-
Texas Longhorn, CTLR	20	0.78	-	-	0.23	-	-	-	-
Tuli	23	0.96	0.04	-	-	-	-	-	-
Wagyu	24	0.94	0.06	-	-	-	-	-	-
Total	1154	0.782	0.052	**0.054**	0.048	0.031	0.030	-	0.003

^a^HIF2A protein variant allele definitions are shown in Figure 4. A hyphen indicates an allele frequency of zero. ^b^HIF2A protein “variant 3” contains the T606, S610 missense mutations previously associated with pulmonary hypertension ^15^.

**Figure 5.**
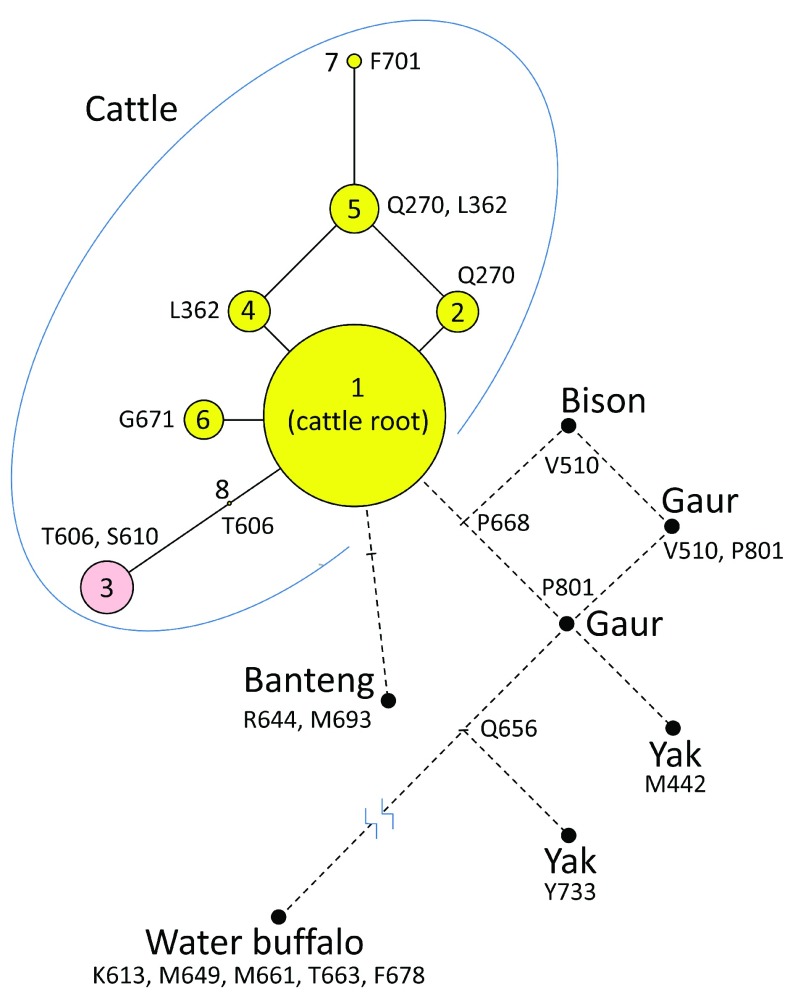
Rooted maximum parsimony phylogenetic tree of HIF2A protein variants found in cattle and closely related species. The cattle HIF2A “variant 1” was used as the reference sequence for comparison with HIF2A from five other species (Methods). Cattle HIF2A residues were highly conserved between these species and only differed at 11 total sites. In “variant 1” the cattle residues at these 11 positions were: V442, K613, T663, L644, M649, R656, M661, L668, F678, V693, H733. For cattle “variants 1” through “8”, the areas of the circles shown are proportional to the variant frequency in the group of 1250 cattle tested. “Variant 3” (pink circle; T606, S610) is identical to that associated with PH in Angus cattle ^15^. The nodes derived from analysis from other species are indicated with a black filled circles and do not represent frequency information.

To determine the most likely phylogenetic root of the HIF2A tree, and thus establish a possible order of mutational events, HIF2A sequences were analyzed in eight individuals from closely related species: from the *Bos, Bison*, and *Bubalus* genera. HIF2A “variant 1” was the likely ancestral root, based on its similarity to HIF2A from the most closely related species ( Figure 5). Thus, the S610 mutation likely occurred on the T606 haplotype and is the more recent mutation of the two. Identifying breeds and individuals that have the HIF2A T606 allele provides the opportunity for future comparisons of the relative effects of T606 alone (“variant 8”), or in combination with S610 (“variant 3”).

**Figure 6.**
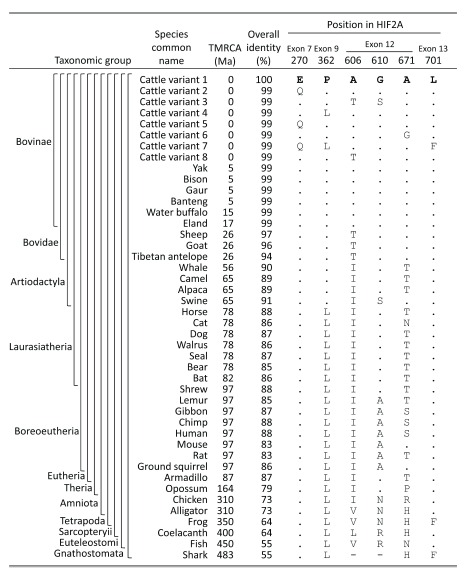
Evolutionary comparison of HIF2A residues from six variant sites. HIF2A sequences from a representative subset of 37 species from the Gnathostomata superclass were deduced from WGS or downloaded from GenBank. Abbreviations and symbols are as follows: TMRCA, estimated time to most recent common ancestor in millions of years ^30^; letters, IUPAC/IUBMB codes for amino acids; dot, amino acid residues identical to those in cattle HIF2A “variant 1”; dash, polypeptide region missing in shark HIF2A.

The highly conserved HIF2A amino acid residues across vertebrates provides insight into the potential impact of missense mutations in cattle, because invariant residues tend to be critical for protein function. The 870 amino acid sequence of cattle HIF2A is highly similar to those from sheep, whale, human, mouse, and alligator (97, 90, 88, 83, and 73% identity, respectively). Alignment of cattle HIF2A sequences with 70 available species of the Gnathostomata superclass showed that a third of the residues (288 of 870) were perfectly conserved throughout ( Table S6). Of the six HIF2A variant sites identified in cattle, the most conserved residue was glutamate at the E270Q site, which was present in all 70 Gnathostomata tested, 37 of which are shown in Figure 6. The leucine residue of the L701F variant site was less conserved, but still present throughout the Amniota, with the phenylalanine variant being present in the Tetrapoda and higher. The glycine residue at the G610S variant site was conserved in the Laurasiatheria, with the notable exception of S610 in swine, a species known for a marked pulmonary vasoconstrictive response to hypoxia. The proline residue of the P362L variant site was conserved through Cetartiodactyla with leucine present in Perissodactyla and higher. The alanine residue of the A671G variant site was conserved in the Bovidae with threonine and other residues present in Cetartiodactyla and higher. Variant A606T was the least conserved of all the variant sites with the alanine residue only conserved in the Bovinae, and the threonine residue present in other ruminants with isoleucine present in Cetartiodactyla and higher. Based exclusively on the degree of conservation across vertebrate species, the predicted ranking for potentially deleterious *EPAS1* missense mutations in cattle was: E270Q > L701F > G610S > P362L > A671G > A606T. However, the actual impact of these polymorphisms on cattle is dependent on additional factors, some of which are discussed in the next section.

## Discussion

Our primary goals were to create a searchable and publicly viewable genomics resource consisting of 96 sires representing a broad cross section of U.S. beef cattle, and demonstrate its use for identifying missense mutations in *EPAS1*, a bovine gene associated with PH and RHF ^15^. To that end, we first determined the amount of WGS required to achieve accurate diploid genotyping when aligned to the bovine reference genome. Our findings verified that 40 GB of short read, paired-end DNA sequence data from the NextSeq500 instrument, provided an average of 12-fold genome coverage. Accordingly, we used 40 GB as a minimum for each animal, aligned the sequences, and made them available online for downloading or viewing with a custom visualization tool that supports accurate assessment of genetic variation. The average coverage of the beef cattle diversity panel was 14.8-fold and resulted in an average genotype accuracy of approximately 99%. These numbers were consistent with results obtained with 379 Canadian beef and dairy cattle, 65 Swiss dairy cattle, and 96 human WGS datasets, sampled at various coverage levels, and compared to bead array data ^10, 31, 32^.

Surprisingly, the amount of effort required to verify the identity and accuracy of the datasets was nearly equal to that required to produce it. Comparing WGS genotypes from 121, well-distributed, highly-informative reference SNPs was sufficient to identify and correct errors in the datasets. However, larger SNP datasets from commercial DNA microarrays provided an additional level of confidence that was useful. Considering the time and resources invested in collecting the WGS, it seemed prudent to have a significant set of independently obtained reference SNP genotypes available for each animal in the group, and use them to validate each WGS dataset.

An important secondary goal of this project was to provide biomedical veterinary researchers the ability to readily inspect gene mutations reported in one breed, evaluate them in other breeds, and search for any additional mutations that may affect protein structure. A web-based platform was created to achieve this goal, and then tested by analyzing *EPAS1*, a gene where missense mutations had been previously documented. Six missense mutations, including the two that were previously reported to be associated with PH ^15^, were readily identified by viewing the aligned raw sequence. The variants were validated by designing MALDI-TOF MS genetic tests and genotyping a broader population of animals. Determining the haplotype phase of these mutations in a total of 1250 individuals from 46 breeds, resulted in eight predicted HIF2A protein variants, five of which were previously unknown. Comparing HIF2A variant frequencies in the 96-member, 19 breed diversity panel with those of the 1154-member, 46 breed set confirmed that the smaller panel was a good approximation of the larger one (r ^2^ = 99.9). Together, the five newly identified HIF2A variants comprised 16.4% of the total alleles in 46 breeds, and represented a significant proportion of the HIF2A variation in cattle. The MALDI-TOF MS genetic test for *EPAS1* was designed to facilitate future study of these variants, and provide a way to control for *EPAS1* stratification in studies of bovine respiratory diseases. The ability to identify the full range of protein variants in a population is critical for designing studies intended to test the candidate gene’s influence on a trait.

The report by Newman *et al.* ^15^, describing an *EPAS1* T606, S610 gene variant associated with PH, raises intriguing questions about the biological mechanisms leading to disease. Among them is the possibility that only one of the two linked missense mutations is pathogenic. Our findings suggest that the S610 variant would be the more plausible candidate of the two. Five lines of evidence are consistent with this hypothesis. First, the S610 missense mutation appears to have arisen on an existing *EPAS1* T606 haplotype, making it the more recent mutation. Younger mutations in functional genes are more likely to be deleterious that older ones ^33^. Second, the G610 residue is conserved across ruminant species, which is consistent with the removal of deleterious alleles by strong purifying selection. Third, among the Laurasiatheria representatives, swine is the only one besides cattle to have the S610 allele. Swine also develop severe PH when exposed to chronic hypoxia ^34^. Fourth, the T606 residue occurs in sheep and goats which have only mild responses to chronic hypoxia ^34, 35^. Fifth, the T606 residue is also present in Tibetan Antelope, a species that evolved at high-altitude and does not suffer from PH. These observations are consistent with the hypothesis that the S610 missense mutation is pathogenic and causes PH in cattle. Alternatively, both alleles may be required in combination to cause disease, or they may be in genetic linkage with an undiscovered cause of PH. Regardless, the pathogenic mutation hypothesis is testable with appropriate individuals from Chianina and Maine-Anjou cattle breeds, since examples of HIF2A T606, S610 (“variant 3”) and HIF2A T606 (“variant 8”) are present in both breeds.

Of the remaining *EPAS1* missense mutations, E270Q stands out as having the greatest potential for affecting the function of HIF2A. This prediction is based on the observation that the E270 residue was invariant across all 70 vertebrate species evaluated. The E270Q variant is located in the PAS-B domain, the second of two tandemly positioned PAS domains. Mutations in murine PAS-B have been shown to affect the ability of HIF2A to sense chemical signals via ligand binding, and thus stabilize the transcriptionally active heterodimer in response to hypoxia, although the E270 residue was not specifically tested ^36^. *EPAS1* haplotypes encoding Q270 residues accounted for 22% of the total in 80 Holstein sires used in the present study, and could be a potential cause of the PH observed Holstein cattle ^16^. These 80 Holstein cattle were also devoid of the *EPAS1* T606, S610 haplotype associated with PH in Angus cattle. Overall *EPAS1* Q270 alleles are present at a frequency of 8% in U.S. cattle tested (HIF2A variants “2”, “5”, and “7”, Figure 4) and are predicted to have deleterious biological consequences.

Another highly conserved residue was L701, which was invariant through the Amniota. However, the F701 substitution was present only on the Q270 haplotype (“variant 7”) and is located in an interdomain region of HIF2A of unknown function. Moreover, the F701 substitution was only observed in one of 1250 animals tested (Salers sire no. 19999882 in MBCDPv2.9). The WGS for this animal at position chr11:28663897, together with concordant genotypes by MALDI-TOF MS confirmed the authenticity of this SNP. However, its exceedingly low frequency makes it unlikely that this substitution, no matter how disruptive, would have a significant impact on U.S. cattle. The remaining missense mutations P362L and A671G were neither highly conserved in vertebrates, nor located in HIF2A regions of known significance. However, their combined frequency in cattle was not insignificant at nearly 8%. Either of these could potentially affect HIF2A function and thereby influence traits associated with *EPAS1.* The substitution of proline for leucine is a particularly significant change that has been shown to cause functional disruptions in other proteins such as T4 lysozyme and caspase-9 ^37, 38^. However, the impact of this substitution remains unknown.

After more than 20 years of selection for herd sires with low pulmonary artery pressures (PAP), 50% of calf mortalities were still attributed to PH in some high-altitude ranches ^39^. We hypothesize that selection for the most common, ancestral HIF2A sequence will enrich for the most favorable allele for U.S. beef cattle and complement efforts that employ PAP testing. This HIF2A sequence contains residues E270, P362, A606, G610, A671, and L701 (“variant 1”, Figure 4) and has the highest amino acid sequence identity when compared to HIF2A from yak, gaur, banteng, and bison. The frequency of “variant 1” was 100% in the Brahman, Brown Swiss, Indu Brazil, Nelore, and mini-zebu breeds; greater than 90% in Ankole-Watusi, Beefmaster, Brahmousin, Devon, Marchigiana, Piedmontese, Romagnola, Simmental, Tuli, Wagyu; and 78% overall. Thus, if employed, selection for HIF2A “variant 1” would not be an exercise in introgression, but rather an effort to remove a minority of potentially deleterious alleles. This may help reduce the overall incidence of PH and the problems associated with it in cattle.

## Conclusion

In summary, the WGS resources described here are suitable for use in identifying and decoding gene variants in the vast majority of U.S. beef cattle. When applied to *EPAS1*, the findings suggest that there may be deleterious alleles circulating in U.S, in addition to those previously associated with high altitude PH. These resources, including the web interface, underlying sequence data, genetic tests, and the associated information are available to researchers, companies, veterinarians, and producers for use without restriction.

## Data availability

The data referenced by this article are under copyright with the following copyright statement: Copyright: © 2016 Heaton MP et al.

Validated cattle FASTQ files are available in the NCBI SRA under accession numbers SRR4001609-SRR4002095; SRR4004613-SRR4004644; SRR4002950-SRR4003067; SRR4003069-SRR4003073; SRR4003075-SRR4003079; SRR4003081-SRR4003085; SRR4003087-SRR4003094; SRR4003096-SRR4003139; SRR4003141-SRR4003146; SRR4003148-SRR4003152; SRR4003154-SRR4003158; SRR4003160-SRR4003164; SRR4003166-SRR4003170; SRR4003172-SRR4003177; SRR4003179-SRR4003182; SRR4003184-SRR4003188; SRR4003190-SRR4003451; SRR4004645-SRR4004679; SRR4004680-SRR4004734; SRR4004736-SRR4004891; SRR4004893-SRR4004920; SRR4004922-SRR4004948; SRR4004950-SRR4004982; SRR4004991-SRR4004992; SRR4004994-SRR4004997; SRR4005006-SRR4005012; SRR4005021-SRR4005026; SRR4005044-SRR4005048; SRR4005057-SRR4005062; SRR4005071-SRR4005195. The data have also been deposited with links to BioProject accession number PRJNA324822 in the NCBI BioProject database ( https://www.ncbi.nlm.nih.gov/bioproject/).

FASTQ files produced from closely related species are available in the NCBI SRA under accession numbers SRR4035250-SRR4035309 and are associated with BioProject accession number PRJNA325061.

In addition, access to the aligned sequences is available via USDA internet site: http://www.ars.usda.gov/Services/Docs.htm?docid=25585. Download access to the .BAM files is available at the Intrepid Bioinformatics site: http://server1.intrepidbio.com/FeatureBrowser/customlist/record?listid=7686214634.

## Supplementary material

**Table S1.** Features of 121 USMARC bovine parentage SNPs.

Click here to access the data

**Table S2.** Summary WGS statistics and reference genotypes for 121 parentage SNPs with the USMARC Beef Diversity Panel Version 2.9.

**Table S3.** Source information for individuals from Bovinae species mapped to the cattle UMD3.1 reference assembly.

**Table S4.** MALDI-TOF MS assay design for six missense mutations in bovine *EPAS1*.

**Table S5.** HIF2A protein variant diplotypes in 1154 animals from 46 breeds of U.S. cattle.

**Table S6.** Alignment of HIF2A sequences from Gnathostomata species.

**Figure S1.** Screen image of IGV software displaying *EPAS1* E270Q genotype data for eight cattle.

**File S1.** VCF file of 772,990 SNP variants used in comparing WGS genotypes to those from a high-density bead array.
