# Supplementary figures and images for: Using diverse U.S. beef cattle genomes to identify missense mutations in EPAS1, a gene associated with pulmonary hypertension
Source: F1000Res. 2016 Oct 5;5:2003. Originally published 2016 Aug 16. [Version 2] doi: 10.12688/f1000research.9254.2 (PMC5040160; doi:10.12688/f1000research.9254.2)

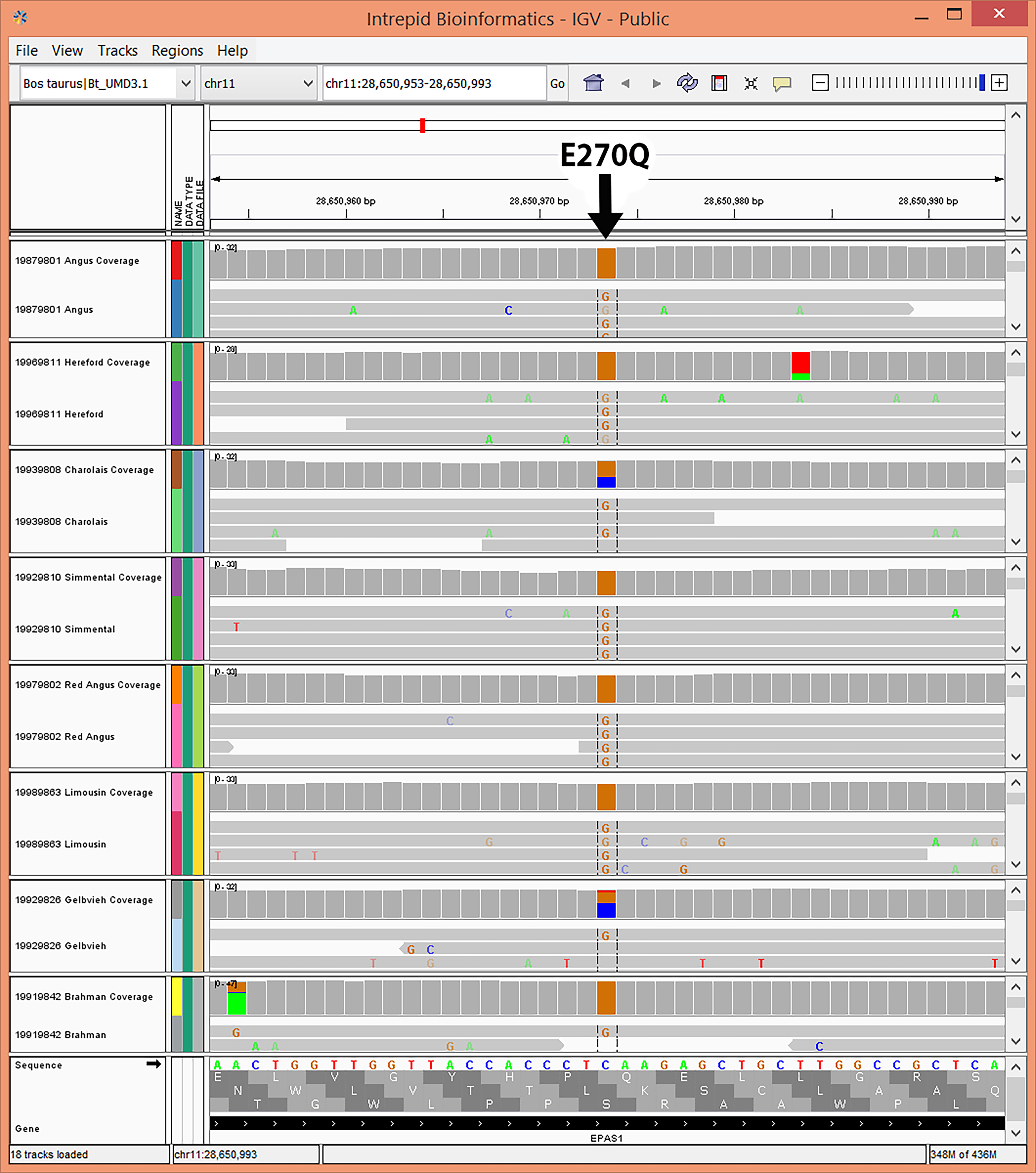

Supplement: Supplementary file 6 [file f1000research-5-10467-s0004.tgz › 497de621-aa9d-4355-8737-5920c5505f03.tif]
