# Supplementary material for: Using diverse U.S. beef cattle genomes to identify missense mutations in EPAS1, a gene associated with pulmonary hypertension
Source: F1000Res. 2016 Oct 5;5:2003. Originally published 2016 Aug 16. [Version 2] doi: 10.12688/f1000research.9254.2 (PMC5040160; doi:10.12688/f1000research.9254.2)
Supplement: Peer review history

# Peer review: Referee response for version 1

**DOI:** 10.5256/f1000research.9962.r16383

**Published:** 2016-09-23

**Authors:** Matthew C. McClure

**Affiliations:** 1 Irish Cattle Breeding Federation, Bandon, Ireland

Overall a very nice paper on *EPAS1* and high-altitude pulmonary hypertension (HAPH). Only time will tell if the more important outcome of this paper is the determination that the G610S alleles likely the causative mutation for HAPH (over A606T), that multiple *EPAS1* alleles could together cause/impact some version of HAPH, or that there is finally a public database to compare whole genome sequence data between studies. As cattle producers and breed associations move to use more genetic tests to minimize genetic variants that cause economic loss in the breed the first 2 points above will be very useful, especially with “50% of calf mortalities… attributed to PH in some high-altitude ranches”, as noted in the manuscript. For researchers studying other genetic diseases the latter point will be more important as such a public database is needed to help filter out non-causative alleles. Currently such researchers have had to turn to other scientists that have their own WGS databases, or to semi-private database like the 1000 bull dataset where you must be a member to access the data. In the past I personally have had to use the former option and was grateful that the bovine community is full of gracious scientists that would help.

 This is a very well written paper and was a pleasure to review.

 Overall I do approve of the manuscript to be indexed and advise only minor edits and some suggestions listed below.

 Suggestions:

1. In the abstract you state that the “existing bovine WGS dabases… diversity in US beef cattle”.  Suggest this be changed to global beef cattle or similar as this issue is not confined to the USA.
2. Page 3, 4 ^th^ paragraph:  While it’s a semi-private WGS dataset, given its size, I do recommend including the 1000 Bulls project when talking about population-scale WGS data.  You could mention that it’s there but not publicly available which is one of the main advantages your WGS data has as reported in the manuscript

 

  Recommended:

1. Introduction: You state that “114 Mendelian traits with known causative mutations”  and reference a 2014 paper. I advise updating that number as OMIA lists (23/9/2016) that 130 Mendelian disorders have their causative mutation known in cattle. I’m fine if you keep the reference you have and include a OMIA reference as your reference is an excellent review paper.
2. Table 1: You present a large amount of data about each variant and this is wonderful as so many publications would only list one or two of your columns. I would like to see the flanking sequence be increased from 5bp per side to 15bp. Ensemble returns 0 hits for all 6 of your flanking sequences with BLASTing against the UMD3.1 assembly. Increasing it to my recommended length allows BLAST to find unique placements of the sequence. For future researchers to be able to test for disease causing variants having minimal information listed in publication makes it a challenge to make sure you are correctly designing diagnostic probes. I speak from experience in designing diagnostic probes for the IDB cattle chip used in Ireland.
3. Table 3 appears to be only a subset of Table 4 with no additional information. Recommend removing Table 3 and only using Table 4. If you have a justifiable reason for keeping Table 3 than advise listing the breeds in alphabetical order and not in variant 3 frequency order.
4. Table 4. In this you show no animals having the variant 7 allele, yet in the 5 ^th^ paragraph on page 14 you state that “the F701 substitution was only observed in one of the 1250 animals tested (Salers,…)". Please include the frequency of this allele in the Salers row in Table 4.
5. Figure 5: Why is node ‘3’ in grey?  It's not stated in the Figure.
6. For multiple Supplementary tables:  Advise including this information from Table 1: cIUPAC/IUBMB ambiguity codes used for nucleotides:…..   Or stating that IUPAC ambiguity codes are used, right now the reader just have to figure it out that a S means the individual is G/C for that SNP.
7. Table S1: Don’t see why columns C, G, or H are needed: Distance between markers (Mb), Nearest gene (UMD3.1), Distance to nearest gene (kbp), respectively. The rest of the data in the table is excellent and wish more papers provided SNP data at this level. Really like that the ambigious SNP in the flanking sequence are shown in bold and capital. I recommend removing column C, G, and H as they are meaningless as to information on Parentage SNP.
8. Table S2: You need to note that a “A, C, T, or G” shown as the genotype indicates that this is a homozygous SNP. One can figure it out since you also show the IUPAC ambiguous letters for heterozygous genotypes, but it would be better to state.
9. Table S5: Column E is labelled as “ *EPAS1* protein variant diplotype”.  In Table 1, 2, 3 you use the phrase “HIF2A protein variant". I advise using the same here, otherwise you can easily confuse the reader. Especially as HIF2A is the name of the protein coded by the *EPAS1* gene, so if you are referring to protein variants HIF2A is more appropriate than *EPAS1*.

I have read this submission. I believe that I have an appropriate level of expertise to confirm that it is of an acceptable scientific standard.

# Response

**Published:** 2016-09-28

**Authors:** Michael Heaton

**Affiliations:** US Meat Animal Research Center, USA

We have provided point-by-point responses to the reviewer's suggestions and recommendations below:

- *Reviewer suggestion:* “In the abstract you state that the ‘existing bovine WGS dabases… diversity in US beef cattle’.  Suggest this be changed to global beef cattle or similar as this issue is not confined to the USA.”

*Authors response:* We agree and have made this change. 

  

- *Reviewer suggestion:* “Page 3, 4 ^th^ paragraph:  While it’s a semi-private WGS dataset, given its size, I do recommend including the 1000 Bulls project when talking about population-scale WGS data.  You could mention that it’s there but not publicly available which is one of the main advantages your WGS data has as reported in the manuscript”

*Authors response:* We agree. Please note that the first reference provided in this paragraph (Daetwyler *et al.* Nat Genet. 2014; **46**(8): 858–65) is the current published reference for the 1000 bulls project. Their sequence is public and available at http://www.ncbi.nlm.nih.gov/bioproject/PRJNA238491. In addition, the WGS from our article is available for inclusion in subsequent rounds of the 1000 bulls project.

  

- *Reviewer recommendation:  *“Introduction: You state that “114 Mendelian traits with known causative mutations” and reference a 2014 paper. I advise updating that number as OMIA lists (23/9/2016) that 130 Mendelian disorders have their causative mutation known in cattle. I’m fine if you keep the reference you have and include a OMIA reference as your reference is an excellent review paper.”

*Authors response:* The reviewer correctly points out that the number of Mendelian traits is increasing. We have updated the text to reflect the 130 entries currently listed on the OMIA web site: http://omia.angis.org.au/home/.

  

- *Reviewer recommendation: *“Table 1: You present a large amount of data about each variant and this is wonderful as so many publications would only list one or two of your columns. I would like to see the flanking sequence be increased from 5bp per side to 15bp. Ensemble returns 0 hits for all 6 of your flanking sequences with BLASTing against the UMD3.1 assembly. Increasing it to my recommended length allows BLAST to find unique placements of the sequence. For future researchers to be able to test for disease causing variants having minimal information listed in publication makes it a challenge to make sure you are correctly designing diagnostic probes. I speak from experience in designing diagnostic probes for the IDB cattle chip used in Ireland.”

 
*Authors response:* We agree. To ensure a robust BLAST result, we have included 50 bp of flanking genomic sequence on each side of the variant SNPs in Table 1 (101 bp total).

  

- *Reviewer recommendation: “*Table 3 appears to be only a subset of Table 4 with no additional information. Recommend removing Table 3 and only using Table 4. If you have a justifiable reason for keeping Table 3 than advise listing the breeds in alphabetical order and not in variant 3 frequency order.”

 
*Authors response: *We have deleted Table 3 and have adjusted the text accordingly.

  

- *Reviewer recommendation: “*Table 4. In this you show no animals having the variant 7 allele, yet in the 5 ^th^ paragraph on page 14 you state that “the F701 substitution was only observed in one of the 1250 animals tested (Salers,…)". Please include the frequency of this allele in the Salers row in Table 4.”

*Authors response: *Table 4 contains genotypes for 1154 animals, including 23 Salers. None of these animals have the F701 variant. There are an additional five Salers in the 96-member beef cattle diversity panel. One of those five Salers has the allele. We have reworded the sentence on page 14 to clarify: “Moreover, the F701 substitution was only observed in one of 1250 animals tested (Salers sire no. 19999882 in MBCDPv2.9).”

  

- *Reviewer recommendation: *Figure 5: Why is node ‘3’ in grey?  It's not stated in the Figure.

 
*Authors response: *“Variant 3” (pink circle; T606, S610) is identical to that associated with PH in Angus cattle. We have added this sentence to the figure legend.

  

- *Reviewer recommendation: *“For multiple Supplementary tables: Advise including this information from Table 1: cIUPAC/IUBMB ambiguity codes used for nucleotides:…..   Or stating that IUPAC ambiguity codes are used, right now the reader just have to figure it out that a S means the individual is G/C for that SNP.

*Authors response: *We agree and will add this information as footnotes to Supplementary Material Tables S2 and S5.

  

- *Reviewer recommendation: *“Table S1: Don’t see why columns C, G, or H are needed: Distance between markers (Mb), Nearest gene (UMD3.1), Distance to nearest gene (kbp), respectively. The rest of the data in the table is excellent and wish more papers provided SNP data at this level. Really like that the ambigious SNP in the flanking sequence are shown in bold and capital. I recommend removing column C, G, and H as they are meaningless as to information on Parentage SNP.”

*Authors response: *The information in Column C (genomic spacing) is an important feature of parentage markers since close proximity between two markers reduces the likelihood of meiotic recombination and thus affects the power for parentage assignment. However, the SNPs distance can be derived from UMD3.1 position so we have removed this redundant column. Regarding the “Distance to nearest gene” column, many parentage SNPs are in genes and thus the gene name is sometimes used as an identifier for parentage SNP.  Because the parentage SNPs are heavily used on bead arrays and in stand-alone assays, their proximity to genes and their location in a gene is information that gets used quite a lot. For these reasons, we prefer not to delete these columns because this information is tedious to extract from other sources.

  

- *Reviewer recommendation: *“Table S2: You need to note that a “A, C, T, or G” shown as the genotype indicates that this is a homozygous SNP. One can figure it out since you also show the IUPAC ambiguous letters for heterozygous genotypes, but it would be better to state.”

*Authors response: *We agree and have added this sentence as a footnote to Table S2: “Homozygous genotypes are listed as a single letter corresponding to the nucleotide at those sites, whereas, IUB ambiguity codes denote heterozygous genotypes those sites: Y = C/ T; R = A/G; M = A/C; K = G/T; W = A/T; S = C/G.”

  

- *Reviewer recommendation: * “Table S5: Column E is labelled as “ *EPAS1* protein variant diplotype”.  In Table 1, 2, 3 you use the phrase “HIF2A protein variant". I advise using the same here, otherwise you can easily confuse the reader. Especially as HIF2A is the name of the protein coded by the *EPAS1* gene, so if you are referring to protein variants HIF2A is more appropriate than *EPAS1”*

*Authors response: *We agree and have change the heading for Column E to “HIF2A diplotype.”. We have also added this footnote to cell G6: “Homozygous genotypes are listed as a single letter corresponding to the nucleotide at those sites, whereas, IUB ambiguity codes denote heterozygous genotypes those sites: Y = C/ T; R = A/G; M = A/C; K = G/T; W = A/T; S = C/G.   Genotypes listed as "O" indicate missing information.”

# Peer review: Referee response for version 1

**DOI:** 10.5256/f1000research.9962.r15711

**Published:** 2016-08-30

**Authors:** Joseph M. Neary

**Affiliations:** 1 Department of Animal and Food Sciences, Texas Tech University, Lubbock, TX, USA

Heaton *et al.* are to be commended for a thorough description of a novel approach to discovering bovine protein variants *in silico*. Further, the authors demonstrate the application of their newly created and publicly accessible mapped genomes of 19 beef breeds by identifying protein variants encoded by *EPAS1*, a gene recently associated with susceptibility to pulmonary hypertension (PH) in cattle at altitudes of 5,200-7,850 ft. (Newman *et al.,* 2015).

 The authors also provide an excellent discussion of the plausible *in vivo* effects of the amino acid variants encoded by *EPAS1*. Of particular interest, is the different variant frequencies reported among the various breeds (Figure 4; Table 4). Interestingly, Holstein cattle are susceptible to hypoxia-induced PH and serve as a favorable animal model of the equivalent human condition; yet, as discussed by the authors, PH in Holstein cattle may be due to an amino acid variant (Q270) that differs from S610 variant that contributes to PH susceptibility in Angus cattle. This demonstrates the importance of a having publicly searchable set of mapped genomes for popular breeds of U.S. cattle.

 Minor revisions:

1. Third paragraph, first line of Introduction: remove conjunction (“However”) from the start of the sentence and place mid-sentence (suggestion only). This may improve sentence flow.
2. I am uncomfortable with the term “high-altitude pulmonary hypertension” because it implies that this physiological phenomenon is only problematic in high altitude regions when, in fact, it occurs at even moderate altitudes in both dairy and beef breeds of cattle (Malherbe *et al.,* 2012; Neary *et al.,* 2016). The Newman study was performed on measurements collected at 5,200-7,850 ft., which in my opinion, is more consistent with moderate than high altitude. “High-altitude” also implies that hypobaric hypoxia is the sole etiology; however, as you discuss, other causes of hypoxia and inflammation, such as respiratory disease, may also be involved. The term pulmonary hypertension (PH) ought to be sufficient. 

I have read this submission. I believe that I have an appropriate level of expertise to confirm that it is of an acceptable scientific standard.

## References

[1] NewmanJHHoltTNCoganJDWomackBPhillipsJALiCKendallZStenmarkKRThomasMGBrownRDRiddleSRWestJDHamidR: Increased prevalence of EPAS1 variant in cattle with high-altitude pulmonary hypertension. *Nat Commun*.2015;6: 10.1038/ncomms7863 6863 10.1038/ncomms7863 25873470PMC4399003

[2] MalherbeCRMarquardJLeggDECammackKMO'TooleD: Right ventricular hypertrophy with heart failure in Holstein heifers at elevation of 1,600 meters. *J Vet Diagn Invest*.2012;24(5) : 10.1177/1040638712453580 867-77 10.1177/1040638712453580 22914818

[3] NearyJMBookerCWWildmanBKMorleyPS: Right-Sided Congestive Heart Failure in North American Feedlot Cattle. *J Vet Intern Med*.30(1) : 10.1111/jvim.13789 326-34 10.1111/jvim.13789 26547263PMC4913666

# Response

**Published:** 2016-09-28

**Authors:** Michael Heaton

**Affiliations:** US Meat Animal Research Center, USA

We agree with the reviewer’s suggestions and have made these changes. Thus, we have removed “high altitude” as a modifier of “pulmonary hypertension” in the title and throughout the article.
